# Cryo-EM Structures and AlphaFold3 Models of Histamine Receptors Reveal Diverse Ligand Binding and G Protein Bias

**DOI:** 10.3390/ph18030292

**Published:** 2025-02-21

**Authors:** Anqi Chen, Chenxi Su, Zisu Zhang, Haitao Zhang

**Affiliations:** The Second Affiliated Hospital of Zhejiang University School of Medicine, Research Center for Clinical Pharmacy, Key Laboratory of Neuropharmacology and Translational Medicine of Zhejiang Province, State Key Laboratory of Advanced Drug Delivery and Release Systems, Institute of Pharmacology and Toxicology, College of Pharmaceutical Sciences, Zhejiang University, Hangzhou 310058, China

**Keywords:** histamine receptors, ligand-binding pocket, activation mechanism, G protein bias, cryo-EM, AlphaFold3

## Abstract

**Background:** The four subtypes of G protein-coupled receptors (GPCRs) regulated by histamine play critical roles in various physiological and pathological processes, such as allergy, gastric acid secretion, cognitive and sleep disorders, and inflammation. Previous experimental structures of histamine receptors (HRs) with agonists and antagonists exhibited multiple conformations for the ligands and G protein binding. However, the structural basis for HR regulation and signaling remains elusive. **Methods:** We determined the cryo-electron microscopy (cryo-EM) structure of the H4R-histamine-Gi complex at 2.9 Å resolution, and predicted the models for all four HRs in the ligand-free apo and G protein subtype binding states using AlphaFold3 (AF3). **Results:** By comparing our H4R structure with the experimental HR structures and the computational AF3 models, we elucidated the distinct histamine binding modes and G protein interfaces, and proposed the essential roles of Y^6.51^ and Q^7.42^ in receptor activation and the intracellular loop 2 (ICL2) in G protein bias. **Conclusions:** Our findings deciphered the molecular mechanisms underlying the regulation of different HRs, from the extracellular ligand-binding pockets and transmembrane motifs to the intracellular G protein coupling interfaces. These insights are expected to facilitate selective drug discovery targeting HRs for diverse therapeutic purposes.

## 1. Introduction

Histamine, as an endogenic biogenic amine, plays a central role in the immune system, gastrointestinal digestive system, central nervous system (CNS), and periphery nervous system, orchestrating numerous pathophysiological processes such as itch, allergy, gastric acid secretion, neurotransmission, and immune responses [1,2]. The four histamine receptor subtypes (H1R-H4R) couple to their specific G proteins and mediate different physiological effects [3]. H1R predominantly couples to Gq, as the target for the anti-allergy treatments [4]. H2R couples to Gs, whose antagonist cimetidine has been used for gastrohelcosis therapy [5]. H3R couples to Gi [2] in the CNS [2,6], and its antagonist pitolisant has been approved for treating narcolepsy and obstructive sleep apnea [6].

H4R preferentially couples to Gi [2] in the immune system, blood vessels, nerves, and enterocytes, and is involved in the expression and release of chemokines and cytokines [7,8]. As a promising target for anti-inflammatory and immunologic reactions [8,9,10,11,12,13], H4R regulates immunologic diseases, including atopic dermatitis, urticaria, allergic asthma, and rheumatoid arthritis (RA) [14,15,16]. Recently, the H4R antagonist toreforant was withdrawn from Phase II trials for RA and eosinophilic asthma due to a lack of efficacy and elusive structure-activity relationship (SAR) studies [17,18]. Additionally, H4R expression is markedly diminished in tumor tissues, and emerging evidence has suggested the anti-tumor and analgesic potentials of H4R agonists in immunodeficient hosts and cancer models [19,20,21,22,23,24].

H4R could also signal to Gq with a slightly lower efficacy (pEC_50_/Gq = 6.8 vs. pEC_50_/Gi = 7.1) [25]. Recently, the cryo-electron microscopy (cryo-EM) structures of H4R-histamine-Gq (PDB ID: 7YFC, as H4R-Gq) and H4R-histamine-Gi (PDB ID: 8YN9/8JXT, as H4R-Gi-1 and H4R-Gi-2) have been reported using different protein engineering with loop truncations, chimera G proteins, dominant negative Gi, and NanoBiT strategies to improve the protein stability [13,26,27]. Surprisingly, the histamine and G proteins exhibited different binding modes in these experimental structures. The primary amine moiety of histamine formed a salt bridge with D94^3.32^ in the H4R-Gq structure [13], while in the two H4R-Gi structures, hydrogen bonds (HBs) were observed between the imidazole group of histamine and D94^3.32^ [26,27]. On the H4R-G proteins interfaces, an HB between D111^3.49^ and Y122^34.5^ was seen in the H4R-Gi-1 structure [27] but was absent in the H4R-Gi-2 structure [26]. Thus, the molecular mechanisms underlying the histamine binding and G protein signaling pathways mediated by H4R remain elusive. Gi signaling is generally associated with the inhibition of cAMP production, while Gq signaling tends to stimulate phosphoinositide turnover and calcium release [28]. Deciphering the precise conformational transitions of H4R during ligand engagement and signal transduction could facilitate the rational design of selective therapeutics targeting either the Gq- or Gi-mediated signaling cascades for immune-related treatments such as allergic disorders and autoimmune conditions.

Structural biology has undergone a profound revolution driven by advances in artificial intelligence (AI) [29,30,31]. AlphaFold3 (AF3) [32,33] has demonstrated remarkable capabilities in predicting the structures of protein complexes, such as the active G protein-coupled receptor (GPCR)-G protein states. Whereas, its predecessor AlphaFold2 (AF2) primarily focused on individual protein structures in the apo states [32,33,34]. Recent studies have highlighted that AF3 outperforms AF2 in GPCR modeling, particularly in predicting the overall backbones [34]. This capability is particularly valuable for modeling GPCR structures in the absence of experimental data, such as the apo state or non-canonical GPCR signaling complexes.

In this study, we determined a new cryo-EM structure of H4R-histamine-Gi at 2.9 Å resolution. To further elucidate the comprehensive mechanisms underlying the histamine recognition, receptor activation, and G protein bias for all four histamine receptors (HRs), we applied AF3 to predict the HR structures in both the ligand-free apo states and G protein coupling states to mitigate the effects of protein engineering on the structure determination. Based on the experimental structures and computational models of the four HRs, we proposed comprehensive mechanisms for the ligand recognition, receptor activation, and G protein bias.

## 2. Results

### 2.1. Cryo-EM Structures Revealed Diverse Histamine Binding in H4R

**Figure 1:**
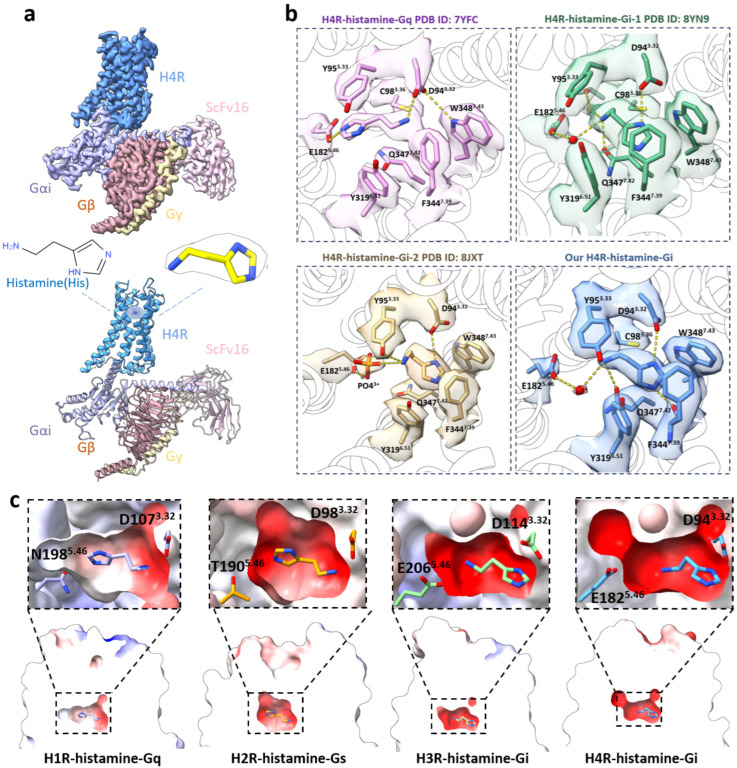
Diverse histamine binding modes in the cryo-EM structures of HRs-histamine. (**a**) Cryo-EM density maps (top) and corresponding structures (bottom) of the H4R-histamine-Gi complex solved in this study. (**b**) Cryo-EM density maps of the respective histamine and ligand-binding pocket (LBP) residues in different H4R-histamine-G protein structures. The primary amine moiety of histamine formed a salt bridge with D94^3.32^ in the H4R-Gq structure, while in the H4R-Gi structures, hydrogen bonds (HBs) were observed between the imidazole group of histamine and D94^3.32^. (**c**) The electrostatic surface of LBPs among the four HRs, from left to right panels: H1R-H4R. The histamine adopted opposite orientations in H3R/H4R from H1R/H2R, probably mediated by the negatively charged residue D^5.46^ in H3R/H4R.

Our cryo-EM structure of H4R-histamine-Gi was determined at 2.9 Å resolution, with unambiguous modeling of H4R, histamine, and Gi proteins (Figure 1a, Figures S1 and S2). The imidazole group of histamine exhibited extensive contacts through π–π stacking (π–π stacking usually occurs between the aromatic residues or compounds with conjugated structures) and hydrophilic interactions with the surrounding residues, consisting of D94^3.32^, C98^3.36^, Y319^6.51^, F344^7.39^, Q347^7.42^, and W348^7.43^ (superscripts denote Ballesteros–Weinstein numbering for GPCRs [35], in which the most conserved residues among all GPCRs are assigned the position 50, and the remaining residues in the helices are numbered relative to this conserved position, with residues closer to the N-terminus given lower numbers). The Nπ atom formed a conserved salt bridge with D94^3.32^ and was further stabilized by W348^7.43^ with π-π packing, enhancing the binding of the imidazole ring of histamine [36]. D^3.32^ and W/Y^7.43^, which are highly conserved in the ligand-binding pockets (LBPs) across the four HRs and other aminergic receptors, routinely form the intermolecular HBs [37] (Figure S3). Additionally, an HB was formed between the Nτ atom of histamine and F344^7.39^. A steep loss was shown in the transforming growth factor (TGF)-α shedding assays and radioligand-binding experiments of the H4R mutants, including D94^3.32^A, F344^7.39^A, and W348^7.43^A [13,26], highlighting the critical roles of these residues in the histamine recognition.

The ethylamine moiety of histamine exhibited extensive HBs and polar interactions in the sub-pocket surrounded by Y95^3.33^, C98^3.36^, E182^5.46^, W316^6.48^, and Q347^7.42^ (Figure 1b), among which, Q347^7.42^ served as a bridge mediating the interaction between histamine and the conserved toggle switch. The Q347^7.42^A mutant resulted in a 14.9-fold decrease in the activity in the TGF-α shedding assays [13]. These findings underscore the essential role of Q347^7.42^ in histamine recognition. The water-mediated HB between the histamine and E182^5.46^ was observed in our structure (Figure 1b). Indeed, alanine substitution of E182^5.46^ was found to abolish the histamine binding, despite the 4.1 Å distance between E182^5.46^ and the cationic amine moiety of histamine [13,38].

The histamine in our structure showed some similar conformations to the previously reported H4R-Gi-1 structure, where the Nπ atom formed a conserved salt bridge with D94^3.32^, and E182^5.46^ stabilized the conformation of the primary amine group of histamine via a water-mediated HB (Figure 1b) [27]. However, the HB between the Nτ atom of histamine and F344^7.39^ observed in our structure was absent in the H4R-Gi-1 structure (Figure 1b) [27]. The conserved salt bridge and HB provide guidance for improving the receptor-ligand interactions in drug design. The conserved interaction suggested that the salt bridge and HBs formed by the histamine and D94^3.32^, E182^5.46^, F344^7.39^, Q347^7.42^, and W348^7.43^ might be key features of the histamine or similar ligands binding to H4R. Drugs designed to mimic the histamine’s binding mode in H4R could exploit those interactions, especially for the compounds that aim to mimic histamine’s natural effects.

In the H4R-Gq structure, the ethylamine of histamine formed a salt bridge with D94^3.32^, while in our H4R-Gi structure, it was the Nπ atom of histamine that formed a conserved salt bridge with D94^3.32^, which exhibited the opposite conformation of histamine in the two structures (Figure 1b) [13]. In the H4R-Gi-2 structure, a phosphate molecule was found in the LBP, which served as an HB donor, stabilizing the conformations of E182^5.46^ and the primary group of histamine through the salt bridge and HB (Figure 1b) [13].

A comparison of our H4R-histamine-Gi structure with previously determined H1R-histamine-Gq (PDB ID: 7DFL [39]), H2R-histamine-Gs (PDB ID: 8POK [40]), and H3R-histamine-Gi (PDB ID: 8YN5 [27]) structures revealed that the histamine-binding modes could be categorized into two patterns, namely, H1R/H2R-like and H3R/H4R-like (Figure 1c). The H1R/H2R LBPs are composed of both negatively charged and uncharged residues, while the H3R/H4R LBPs are predominantly negatively charged. This difference was attributed to the variations at position 5.46, where asparagine and threonine are present in H1R/H2R, while aspartic acid is present in H3R/H4R (Figure 1b). Owing to the stronger positive charge of the primary amine moiety than the imidazole group of histamine, the orientation of histamine was more defined in H1R/H2R due to the partially negatively charged environment in their LBPs (Figure 1c). In contrast, the histamine adopted the opposite orientations in H3R/H4R, probably mediated by the strongly zincative residue D^5.46^ (Figure 1c). These observations might explain why H1R/H2R bind histamine at a low affinity of micromolar level, while H3R/H4R bind histamine at a high affinity of nanomolar level [2]. Additionally, the other aminergic receptors such as the dopamine D1R [41], muscarinic acetylcholine M1R [42], and serotonin 5HT_1A_R [43] also exhibit the H1R/H2R-like binding pattern of monoamines due to the presence of uncharged residues at 5.46 (Figure S3).

Therefore, the comparative analysis of our H4R-histamine-Gi structure with the previously determined H4R-G protein complexes (PDB ID: 7YFC/8YN9/8JXT [13,26,27]) revealed diverse histamine binding modes, characterized by the histamine orientations and anion- or water-mediated HBs formed between the histamine and E182^5.46^. These structural variations in the histamine binding modes likely arise from the methodological differences in the cryo-EM experiments. A systematic comparison of the four H4R–G protein complexes uncovered that D94^3.32^, E182^5.46^, F344^7.39^, Q347^7.42^, and W348^7.43^ are critical components of LBP (Figure 1b). This conserved architecture provides a structural blueprint for designing the histamine-mimetic therapeutics targeting H4R. The monoamine binding modes among the aminergic receptor family could be categorized into two patterns as the H1R/H2R-like and H3R/H4R-like, which exhibit opposite orientations of the imidazole group, largely stemming from the electrostatic variations at position 5.46. These findings broaden the understanding of the monoamine recognition mechanisms within the aminergic receptor family.

### 2.2. Cryo-EM Structures Revealed Diverse G Protein Binding in H4R

**Figure 2:**
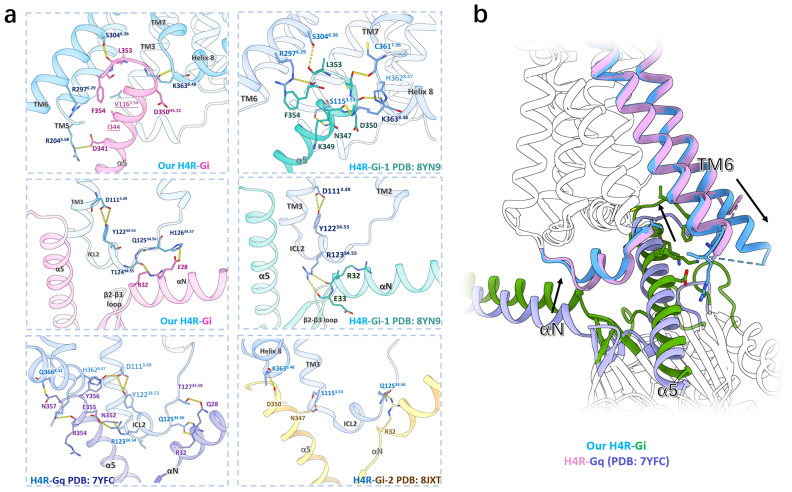
Diverse G protein interfaces in the cryo-EM structures of H4R-histamine-G proteins complexes. (**a**) Comparison of the four cryo-EM structures of H4R-Gi and H4R-Gq at the G proteins interfaces. Top two for the α5 helix interface and bottom four for the ICL2 interface. Yellow dashed lines indicate HBs and blue dashed lines indicate cation-π interactions. Residues for HBs and HIs are depicted as bold and underlined, respectively. Cryo-EM structures of H4R-G protein complexes revealed diverse G protein binding modes in H4R (**b**) Comparison of our H4R-Gi structure (blue for H4R and green for Gi) and the H4R-Gq structure (PDB ID: 7YFC, pink for H4R and purple for Gq) revealed the elevation of the Gi protein coupled to H4R, especially the αN helix and α5 helix, and the prolonged TM6 helix.

At the G protein binding surface from our H4R-Gi structure, multiple HBs and hydrophilic interactions were observed in the α5 helix and ICL2 interface. In the α5 helix interface, S304^6.36^, K363^8.48^, and R204^5.68^ of H4R formed HBs with L353, D350, and D341 of α5 helix, respectively (Figure 2a). The HB between K363^8.48^ and D350 of Gi in the α5 helix interface was also observed in other H4R-Gi structures (Figure 2a) [26,27]. In the ICL2 interface of our structure, Q125^34.56^ and H126^34.57^ of ICL2 formed HBs with R32 and E28 of the Gi αN helix, respectively (Figure 2a).

Unexpectedly, our H4R-Gi structure revealed several interactions different from those in the previously determined H4R-Gi structures [26,27]. The overall root-mean-square deviation (RMSD) between our H4R-Gi structure and the H4R-Gi-2 structure [26] is 2.045 Å, with an RMSD of 1.092 Å for the H4R portion (RMSD can be used to assess the structural similarity or discrepancy between two structures, with RMSD values below 1 Å indicating very similar conformations). In the ICL2 interface of H4R-Gi-2, only three HBs were identified (Figure 2a). In addition to the HBs found in all three H4R-Gi structures, S115^3.53^ and Q125^34.56^ of H4R formed two other HBs in the H4R-Gi-2, with N347 and R32 from the Gi α5 helix and αN helix, respectively (Figure 2a). Notably, the conserved HB established by D111^3.49^ and Y122^34.53^, which was critical for stabilizing the ICL2 conformation and was present in both the H4R-Gi-1 structure and our structure, was absent in the H4R-Gi-2 structure (Figure 2a) [37,40]. These variations might be due to the different protein engineering strategies used for the cryo-EM experiments, which induced conformational changes in H4R and Gi [44].

Our H4R-Gi structure exhibited a similar conformation to the H4R-Gi-1 structure [27], with an RMSD of 0.416 Å globally and 0.307 Å for H4R. In the H4R-Gi-1 structure, six HBs were established in the α5 helix interface, among which, two HBs were also found in the H4R-Gi-1 structure and our structure, formed by S304^6.36^ and K363^8.48^ of H4R with L353 and D350 of the Gi α5 helix, respectively (Figure 2a). In the H4R-Gi-1 structure, R297^6.29^, C361^7.56^, H362^8.47^, and S115^3.53^ of H4R established four other pairs of HBs with F354, C351, D350, and N347 of the Gi α5 helix (Figure 2a). In the ICL2 interface, the HB was stabilized between R123^34.56^ and E33 of the αN-β1 loop, which was missing in our structure (Figure 2a).

The overall RMSD between our H4R-Gi structure and the H4R-Gq structure [13] is 1.068 Å, with an RMSD of 0.748 Å for the H4R portion. In our H4R-Gi structure, from the C-terminus of the Gi α5 helix, the HB was observed between S304^6.36^ of H4R and L353 of Gi (Figure 2a). While, in the H4R-Gq structure, no HB was found between the α5 helix and TM5/TM6 (Figure 2a) [45]. These HBs might contribute to the shift of the Gi α5 helix toward TM5/TM6 and the deeper insertion into the intracellular core in our structure (Figure 2a,b). In addition, a cation-π interaction was established between R297^6.29^ and F354^G.H5.26^ at the C-terminus of the Gi α5 helix in our structure (Figure 2a). Consequently, TM6 extended to H292^6.24^, with nine extra residues compared to the H4R-Gq structure, displaying a pronounced helical extension of 10.8 Å (Figure 2a). The elevation of the α5 helix and αN helix of Gi resulted in an increased buried surface area of the H4R-Gi complex (2446 Å^2^), which is larger than that of the H4R-Gq structure (1823 Å^2^) (Figure 2b).

In all H4R-G protein structures, ICL2 was packed against a cleft established by the αN helix, α5 helix, and β2-β3 loop, primarily through the electrostatic and hydrophobic interactions (HIs) (Figure 2a). This helical conformation of ICL2 in H4R was also observed in other aminergic receptors [40] by the HB between D111^3.49^ and Y122^34.53^ (Figure 2a). In our H4R-Gi complex, additional HBs were formed between H126^34.57^ and E28^αN^, as well as Q125^34.56^ and R32^αN^ (Figure 2a). In contrast, R123^34.54^ from the H4R-Gq formed HBs with both N352^G.H5.19^ and N355^G.H5.22^ in the α5 helix, inducing a shallower binding conformation of the Gq α5 helix (Figure 2a,b).

Therefore, the comparisons of all the H4R-G protein complexes unraveled the diverse binding modes including the α5 helix and ICL2 interface, which might result from the chimeric G proteins explored for the cryo-EM experiments. More interactions were observed in the canonical Gi transducer than Gq, both polar and nonpolar, particularly in the TM5/6, leading to the elevation of the global position of Gi compared to Gq (Figure 2b), thus a more compact conformation was formed in H4R-Gi. These structural arrangements might determine the preferential recruitment of the specific G proteins by H4R.

### 2.3. Histamine Induced Conformational Changes of LBPs Among HRs

We applied AF3 [32,33] to predict the ligand-free apo state models of all four HRs, in which the pTM was over 0.5, which showed high similarity to the true structures [46] (Table 1, Figure S4). The AF3-predicted models achieved a global RMSD of 0.566–1.127 Å compared to the experimental structures in the antagonist-bound inactive state, indicating that AF3 effectively captured the global structural features (Table 1). The LBPs in the predicted models were largely of very high confidence (pLDDT > 90) (Figure S5), which indicated the highest precision category in both the main chains and side chains as the AlphaFold Server Guides; while, the confidence was low in the flexible regions such as the N/C termini, extracellular/intracellular loops, and the distal part of helix 8 (Figure S5).

**Figure 3:**
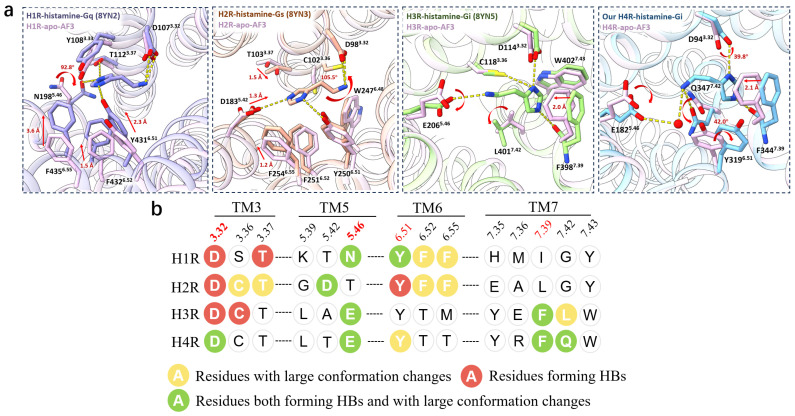
Conformational changes in the LBPs of all four HRs induced by the histamine binding. (**a**) 3D visualization illustrating the LBPs conformational changes from the apo state to the histamine binding state of H1R, H2R, H3R, and H4R, from left to right panels. HBs and polar interactions are depicted as yellow dashed lines, while red dots in the rightmost panel represent water molecules. Notable movements of residues are marked with red arrows. (**b**) Barcodes represent the conformational change patterns of LBP residues induced by the histamine binding in different HRs. Residues forming HBs with histamine are marked with red circles, and residues encountering significant rearrangements upon the histamine binding are highlighted with yellow circles. Green circles stand for both the HBs and large conformational changes. Together, site 3.32 formed an HB with histamine across HRs upon the histamine binding, and sites 5.46 of H1R/H3R/H4R and 5.42 of H2R exhibited remarkable conformational changes upon the histamine binding. Site 6.51 and 7.39 were conserved in forming HBs with histamine for H1R/H2R and H3R/H4R.

Comparison of our H4R-histamine structure with the apo H4R model suggested that the histamine binding induced significant conformational changes in LBP, especially the 2.1 Å movement of F344^7.39^, 42° rotation of Y319^6.51^, and a rotamer flip of E182^5.46^ (Figure 1b and Figure 3a). Mutagenesis of F344^7.39^A, Y319^6.51^A, and E182^5.46^A resulted in substantial decreases in the histamine binding in the radioligand-binding experiments [26], and a steep loss in the TGF-α shedding assays [13], indicating the critical roles of these residues for the histamine binding in H4R. By precisely tuning these interactions, it might either promote or inhibit the specific signaling pathways mediated by H4R, thereby offering a refined approach to therapeutic intervention [26].

Regarding H3R, E206^5.46^, F398^7.39^, and L401^7.42^ exhibited significant conformational changes upon the histamine binding by forming the salt bridge, π–π stacking, and HIs (Figure 3a). Notably, E206^5.46^ could form an HB with histamine in H3R, while in H4R, the HB between E182^5.46^ and histamine was mediated by a water molecule (Figure 3a,b). The alanine mutations of E206^5.46^, F398^7.39^, and L401^7.42^ unequivocally abolished the capability of the Gi activation in the histamine-induced Gi dissociation assays, indicating these residues played vital roles in the histamine binding [47].

Unlike H3R/H4R, the TM7 residues of H1R/H2R did not exhibit notable disparities between the apo and histamine-bound states (Figure 3a). However, in H1R, the aromatic residues of Y431^6.51^, F432^6.52^, and F435^6.55^ moved towards TM3 by 2.3 Å, 1.5 Å, and 3.6 Å, respectively (Figure 3a,b). In addition, N198^5.46^ flipped 92.8° to form an HB with the amine moiety of histamine (Figure 3a). The N198^5.46^A and Y431^6.51^F mutations could completely ablate the histamine-induced H1R activation in the cellular Ca^2+^ assays [39], underscoring the prominence of positions 5.46 and 6.51 for the histamine binding. Surprisingly, D107^3.32^, Y108^3.33^, and T112^3.37^ in TM3, which participated in the HBs or salt bridges with histamine, did not exhibit significant conformational changes (Figure 3a). In H2R, smaller inward movements of the extracellular end in TM6 were observed in Y250^6.51^, F251^6.52^, and F254^6.55^, by 0.7 Å, 0.6 Å, and 1.2 Å, respectively (Figure 3a). While, T103^3.37^ and D183^5.42^ showed larger movements toward histamine by 1.5 Å and 1.3 Å, respectively (Figure 3a). Additionally, C98^3.36^ rotates by 105° to form a polar interaction with the primary amine group of histamine (Figure 3a).

Therefore, the predictions of the LBP in the apo models were highly confident, making analysis based on these models reliable. Comparative analysis of the histamine-bound structures and apo models revealed that significant conformational changes at site 5.46 in H1R/H3R/H4R, and site 5.42 in H2R, accompanied by the formation of HBs upon the histamine binding (Figure 3b). In H1R/H2R, the aromatic residues F^6.52^ and F^6.55^ underwent substantial conformational rearrangements, while in H3R/H4R, the corresponding changes were observed in Y^6.51^ and F^7.39^ (Figure 3b). These differences were likely influenced by the interactions of the imidazole ring of histamine (Figure 3b). Additionally, a unique flipping motion at position 7.42 was observed exclusively in H3R/H4R (Figure 3b). These findings highlighted both the conserved and distinct conformational dynamics within the LBPs of HRs upon the histamine binding. Such insights provide the structural foundation for the rational design of selective drugs, offering new avenues for therapeutic development.

### 2.4. H4R Adopted Unique Activation Mechanisms

**Figure 4:**
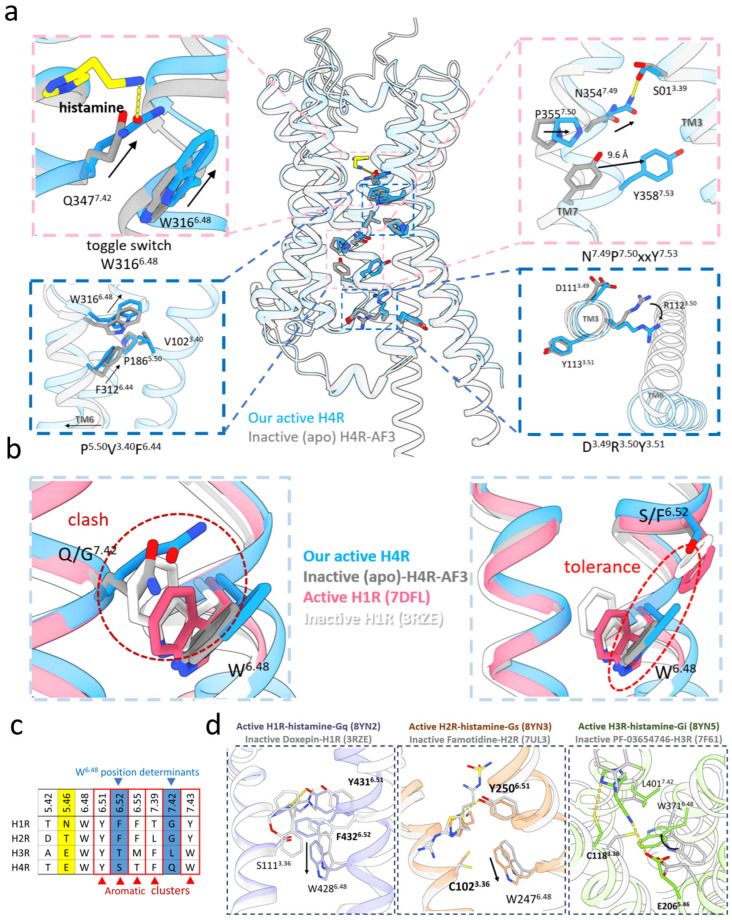
Unique activation mechanism of H4R. (**a**) Structural superposition of the histamine-bound H4R active structure (blue) and apo model (grey) from the side view and enlarged views. The HBs are depicted as yellow dashes between histamine and Q347^7.42^, which in turn attracted W316^6.48^ toward TM3 through π-π stacking. Close-up views highlighted the conformational changes in the conserved microswitches, including the toggle switch, P^5.50^V(I)^3.40^F^6.44^ motif, N^7.49^P^7.50^xxY^7.53^ motif, and D^3.49^R^3.50^Y^3.51^ motif upon the receptor activation. (**b**) Structural superpositions of H1R and H4R in both the active and inactive (apo) states implied that W316^6.48^ adopted a more horizontal conformation in H4R compared to H1R, as the clash of the bulkier side chain of Q347^7.42^, and the tolerance of S320^6.52^ in H4R. (**c**) Sequence alignments revealed the residues critical for the histamine binding and receptor activation, including the inclined position of toggle switch determinants (blue), aromantic clusters (red), and histamine binding orientation determinants (yellow) in HRs. (**d**) Rearrangements of the toggle switch induced by the histamine binding from the antagonist-bound inactive state among HRs are illustrated (from left to right panel: H1R-H3R). Black arrows indicate the conformational changes in the microswitches upon the receptor activation. Residues at positions 5.46, 6.51, and 7.42 acted as the toggle switch helpers for the HR activation since the histamine could hardly interact with the toggle switch directly.

To elucidate the activation mechanism of H4R, our H4R-histamine-Gi structure was superimposed on the AF3-predicted apo model of H4R in the ligand-free apo state (Figure 4a). The H4R apo model exhibited a canonical inactive conformation characterized by a straight TM6 and an open extracellular vestibule (Figure 4a). Upon activation, the H4R-histamine-Gi complex adopted a typical active conformation, including the outward movement of the intracellular end of TM6 and the inward movement of the intracellular tilt of TM7 (Figure 4a).

The toggle switch, which implies the site W^6.48^ and is located in the middle part of the transmembrane bundles in the class A GPCR, bridges the extracellular stimuli and intracellular signaling conduction by conducting a significant conformational change that enables the receptor activation upon the ligand binding [48]. The toggle switch W316^6.48^ of H4R adopted an inclined orientation relative to the lipid bilayer with 65°, in both the histamine-bound active structure and the H4R apo model, different from the typical vertical conformations in H1R/H2R (Figure 4b). Structural superpositions of either the inactive or active states of H1R/H4R revealed the clashes between F^6.52^ of H1R and W^6.48^ of H4R, as well as W^6.48^ of H1R and Q^7.42^ of H4R (Figure 4b,c). Thereby, the oblique orientation of the toggle switch of H4R might stem from the push of the bulkier side chain of Q347^7.42^ and the tolerance of S320^6.52^ in H4R, which were replaced by the smaller glycine and bulkier phenylalanine in H1R and H2R, respectively (Figure 4b,c). Position 7.42 in the aminergic receptors usually adopted glycine, except for the cysteine in the muscarinic acetylcholine receptors, leucine in H3R, and glutamine in H4R, respectively (Figure 4c and Figure S3). Regarding position 6.52, phenylalanine is conserved, except for the aspartate in the muscarinic acetylcholine receptors and certain trace amine receptors, threonine in H3R, and serine in H4R, respectively (Figure 4c and Figure S3). These variations might allow the indole ring of W^6.48^ to adopt a horizontal orientation in H3R/H4R, which share a higher sequence similarity compared to H1R/H2R. Thus, the toggle switch in H3R/H4R displayed a unique conformation in the aminergic receptor family (Figure 4b,d and Figure S3).

The HB between the amino group of histamine and Q347^7.42^ of H4R induced the indole ring of W316^6.48^ to shift upward to TM3 by the π-π packing, further stabilized by E182^5.46^, which was in contrast to the downward movement of toggle switch observed in most other aminergic GPCRs [39,40,41,49] (Figure 4a). This movement stabilized the active conformation of the P^5.50^I(V)^3.40^F^6.44^ motif via the HIs, with F312^6.44^ undergoing a 2.1 Å displacement toward TM5 (Figure 4a). Additionally, S101^3.39^ formed an HB with N357^7.47^, which was associated with the displacement of side chains in the N^7.49^P^7.50^xxY^7.53^ motif, leading to the inward movement of the TM7 end (Figure 4a). For the D111^3.49^-R112^3.50^-Y113^3.51^ motif, the movement of R112^3.50^ toward the center of the intracellular cavity was observed, thus releasing the cytoplasmic end of TM6 from TM3, opening up the transmembrane (TM) cavity to allow the α5 helix of Gi to stretch into the TM core of H4R [48] (Figure 4a). Together, these conformational changes in the microswitch might result in the global motions of the H4R TM bundle, which exhibited the active state in class A GPCRs.

The experimentally determined antagonist-bound inactive structures (PDB ID: 3RZE/7UL3/7F61) were employed to decipher the activation mechanisms of the HRs [6,50,51]. Comparing the histamine-bound active H1R structure (PDB ID: 8YN2 [27]) with the doxepin-bound inactive H1R structure (PDB ID: 3RZE [51]), the imidazole ring of histamine formed an HB with Y^6.51^, resulting in a downward displacement of F432^6.52^ and W428^6.48^, which subsequently led to an outward shift of the intracellular end of TM6 in the active H1R (Figure 4d). The histamine-bound active H2R (PDB ID: 8YN3 [27]) formed an identical HB with Y^6.51^ as that observed in the active H1R (Figure 4d). Mutations of Y^6.51^A and Y^6.51^F totally abolished the H1R/H2R activation in the histamine-induced Gα-Gγ dissociation assays [27] and nuclear factor of activated T-cells response element (NFAT-RE) reporter assays [40], indicating the significant role of position 6.51 in the H1R/H2R activation. The toggle switch of the active H2R moved slightly downward compared to the active H1R, preventing a steric collision through the flipping of the C102^3.36^ side chain from the famotidine-bound inactive H2R (PDB ID: 7UL3 [50]) (Figure 4d). In contrast, W371^6.48^ in the histamine-bound active H3R (PDB ID: 8YN5 [27]) and the PF-03654746-bound inactive H3R (PDB ID: 7F61 [6]) both adopted horizontal orientations, similar to H4R (Figure 4a,d). The W371^6.48^ in the active H3R was stabilized by E206^5.46^, forming π-π stacking with the imidazole group of histamine (Figure 4d). Notably, the alanine mutation of E206^5.46^ completely ablated the capacity of Gi activation in the histamine-induced Gi dissociation assays [47], implying the significance of site 5.46 for the H3R activation. Upon the histamine binding, the rotations of C118^3.36^ and L401^7.42^ side chains provided a space for the W371^6.48^ accommodation from the inactive state to the active state (Figure 4d).

Comparisons of the various HR-histamine structures and toggle switch conformations revealed that the histamine had minimal direct impacts on the toggle switch due to its shallower insertion into LBPs [27], with the concept of “expand to deactivate and squash to activate” [39]. To initiate the conformational rearrangement of W^6.48^ for the activation, the four HRs adopted different mechanisms, in which H1R/H2R employed Y^6.51^, H3R applied E206^5.46^, and H4R relied on Q347^7.42^. The synergistic interactions between the auxiliary residues and toggle switch were reminiscent of the “double toggle switch” L(F)^3.36^-W^6.48^, as previously described in AT1R [52] and GPR84 [53], which played pivotal roles in the receptor activation processes [53,54,55].

Therefore, H4R adopted a more horizontal conformation of toggle switch W^6.48^, due to the push of the bulkier side chain of Q347^7.42^, and the tolerance of S320^6.52^ (Figure 4b), which is unique among the aminergic receptor family (Figure S3). Histamine could hardly interact with the toggle switch directly due to the shallow depth of the LBPs. Thus, the residues at positions 5.46, 6.51, and 7.42 acted as toggle switch helpers for HR activation, highlighting the distinct activation mechanisms of H3R/H4R from H1R/H2R. These findings are expected to provide deep insights into the unique horizontal posture of toggle switch in H4R and the comprehensive activation mechanisms of HRs.

### 2.5. ICL2 Determined G Protein Bias Among HRs

The signaling of H1R-Gq, H2R-Gs, H3R-Gi, and H4R-Gi have been extensively investigated in previous studies [13,26,27,39,40,47]. Their cryo-EM structures revealed that the ICL2s of all four HRs were involved in the respective G protein subtypes binding [39,40,47]. To investigate the G protein subtype preference among HRs, 12 models of the four HRs binding to the three G protein subtypes were generated using AF3 (Figure S4). The pTM scores of all 12 models were above 0.69, which showed high similarity to true structures as higher than 0.5 [46] (Table 1). The ipTM scores were above 0.67, with values below 0.6 indicating a failed prediction as the AlphaFold Server Guides. The AF3-predicted models achieved a global RMSD of 0.669–1.191 Å when compared with the experimental structures, implying that the AF3 models were proficient in capturing the structural characteristics of the complexes (Table 2). The ICL2s in the predicted models were confident (pLDDT > 70, Table 2, Figure S5).

**Figure 5:**
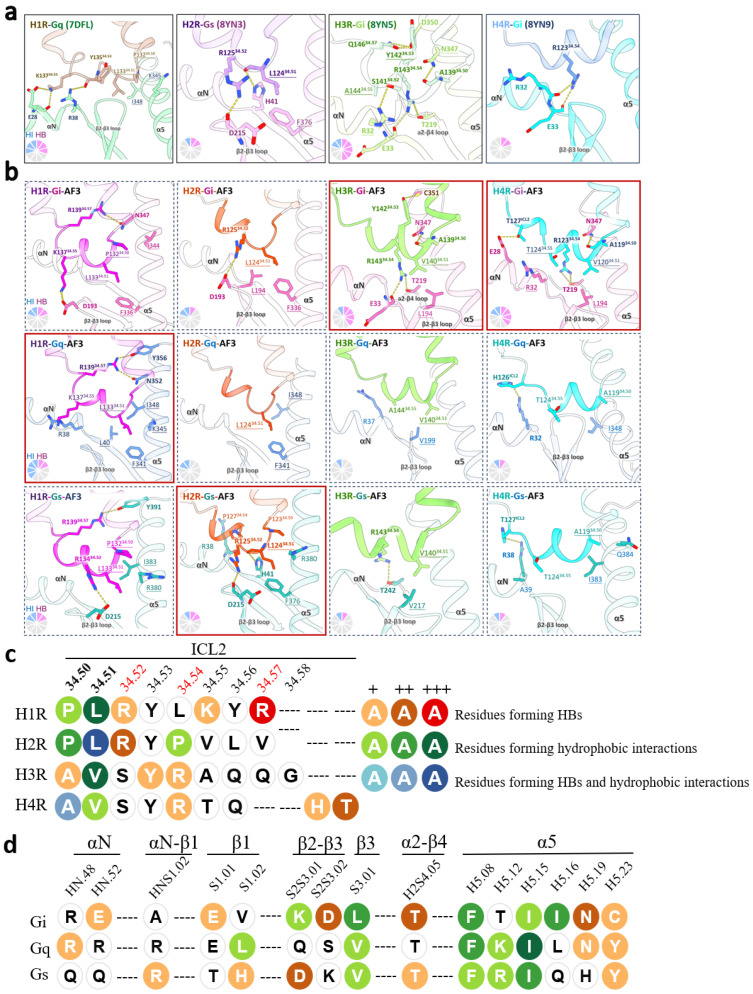
The interactions of ICL2 are the determinants for the G protein preference in HRs. (**a**) 3D visualization illustrating the interface of ICL2 in the experimental structures. From left to right panels: H1R, H2R, H3R, and H4R. HBs, salt bridges, and polar interactions are depicted as yellow dashed lines, with the interacting residues depicted as bold. The underlines represent the residues for the hydrophobic interactions (HIs). The key interactions have been collected as the sector diagram, pink for HBs and blue for HIs. (**b**) 3D visualization illustrating the interfaces of ICL2. HRs with their primary G protein engagements are boxed with red solid lines and other G protein subtypes are marked with dash lines. From left to right panels: H1R, H2R, H3R, and H4R. From top to bottom panels: Gi, Gq, and Gs. ICL2 mediated more interactions including HBs and nonpolar interactions with the primary G protein. (**c**) Barcode representation illustrating the interaction patterns on the ICL2 between the receptor and their respective G proteins in the AF3-predicted models. The nubers of + represent the times of the interactions formed. The darker colors represented the more interactions. The Arg in site 34.52, 34.54 and 34.57 probably formed HBs with HRs in the predicted models. (**d**) Barcode representing the interaction patterns at the G protein interfaces with ICL2 in the AF3-predicted models. Residues for HBs and HIs are indicated by the orange circles and green circles, respectively. Blue circles stand for HBs and HIs. The darker colors represented the more interactions. The αN is the N terminal α helix, the α5 helix is the fifth helix of the G protein, and the helix is denoted as H. The β1 is the first β sheet of G protein, and the sheet is depicted as S. The loop is between the helices or sheets, such as the β2-β3 loop, or the S2S3 loop. The numbering begins at the N-terminus of each helix and progresses toward the C-terminus. The C-terminus of α5 helix exhibited more HBs and the N-terminus of α5 helix displayed more HIs with HRs in the predicted models.

Overall, the predicted models were similar to the experimental structures; however, several specific conformational variations were observed. The experimental structure [40] and computational model of the H2R-Gs showed two identical HBs between L124^34.51^ and H41 of the Gs β1 sheet, as well as R125^34.52^ and D215 of the Gs β2-β3 loop (Figure 5a,b). While in the H1R-Gq and H3R/H4R-Gi [27], the predicted models showed variations from the cryo-EM structures. In the H1R-Gq structure, HBs were formed between E28 of the Gq αN helix and K137^34.55^ of H1R, as well as R38 of the Gq αN-β1 loop and Y135^34.53^ of H1R (Figure 5a) [39], while in the predicted H1R-Gq model, HBs were formed between R139^34.57^ of H1R and N352/Y356 of the Gq α5 helix (Figure 5b). In the H3R/H4R-Gi structures, five and two pairs of HBs were observed, respectively. While in both the H3R/H4R-Gi models, four pairs of HBs were found (Figure 5a,b). Among the five pairs of HBs seen in the H3R-Gi structure, three of which were also predicted, between A139^34.50^ of ICL2 and N347 of the Gi α5 helix, as well as R143^34.54^ of ICL2 with both E33 of the Gi αN-β1 loop and T219 of the Gi α2-β4 loop (Figure 5a,b). Two HBs between Q146^34.57^ of H3R and D350 of the Gi α5 helix, and S141^34.52^ of H3R and R32 of the Gi αN-β1 loop were absent in the computational model (Figure 5a,b). In the H4R-Gi structure [27], only two pairs of HBs were observed between R123^34.54^ of ICL2 and R32/E33 of the Gi αN-β1 loop/β1 sheet, respectively (Figure 2a and Figure 4a). In the H4R-Gi model, R123^34.54^ of ICL2 formed HBs with T219 of the Gi α2-β4 loop instead, and two other pairs of HBs were found between T127 of ICL2 and E28 of the Gi αN helix, and A119^34.50^ of ICL2 and N347 of the Gi α5 helix (Figure 5b). These discrepancies might be caused by the G proteins chimera and mutations, as well as nanobodies used for the cryo-EM experiments [27,39,47].

The ICL2 was embedded in the cleft consisting of the αN helix, αN-β1 loop, β2-β3 loop, and α5 helix (Figure 5a,b). In the predicted models, the α2-β4 loop also participated in occupying the cleft (Figure 5b). In the H1R-Gq model, R139^34.57^ in ICL2 formed two pairs of HBs with N352 and Y356 of the Gq α5 helix, respectively (Figure 5b). In addition, the HIs in ICL2 were also observed to contribute to the H1R-Gq complex formation by L133^34.51^ and L40 of the Gq β1 sheet, and F341/K345/I348 of the Gq α5 helix, respectively (Figure 5b). In the H1R-Gi model, an HB was observed between R139^34.57^ and N347 of the Gi α5 helix (Figure 5b). However, two HIs were observed between L133^34.51^ in ICL2 and F336 of the Gi α5 helix, and P132^34.50^ and I344 of the Gi α5 helix (Figure 5b). The H1R-Gs model showed two HBs between R134^34.52^ and D215 of the Gs β2-β3 loop, and R139^34.57^ and Y391 of the Gs α5 helix (Figure 5b), as well as two HIs between L133^34.51^ and R380 of the Gs α5 helix, and P132^34.50^ and I383 of the Gs α5 helix (Figure 5b). Together, more HIs on the ICL2 interface in the H1R-Gq model compared to the H1R-Gi/Gs models were identified, which might explain that H1R prefers Gq as its primary transducer (Figure 5b).

In the H2R-Gs model, L124^34.51^ and R125^34.52^ of ICL2 formed two pairs of HBs with H41 of the Gs β1 sheet and D215 of the Gs β2-β3 loop, respectively (Figure 5b). The HB between R125^34.52^ of ICL2 and D193 of the Gi β2-β3 loop were also seen in the H2R-Gi model, but no HB was found in the H2R-Gq model (Figure 5b–d). L124^34.51^ of ICL2 formed the HIs with the phenylalanine in position G.H.5.08 (code refers to the universal Gα numbering system [45]) across all G protein subtypes (Figure 5b–d). Additionally, another HI was found in L124^34.51^ of ICL2 and L194 of the Gi β2-β3 loop, and I348 of the Gq α5 helix, respectively. In summary, the H2R-Gs model exhibited more HBs on the ICL2 interface compared to the H2R-Gi/Gq models. This observation suggested a structural preference of Gs for H2R as its dominant transducer (Figure 5b).

The H3R-Gi model exhibited four pairs of HBs, which were established between A139^34.50^ in ICL2 and N347 of the Gi α5 helix, R143^34.54^ and E33 of the Gi αN-β1 loop/T219 of the Gi α2-β4 loop, and Y142^34.53^ in ICL2 and C351 of the Gi α5 helix (Figure 5b). Only one HB was observed in the H3R-Gs model, between R143^34.54^ in ICL2 and T242 of the Gs α2-β4 loop (Figure 5b–d), while no HB was found in the H3R-Gq model (Figure 5b). In different H3R-G protein models, HIs were observed between V140^34.51^ of ICL2 and L/V in position G.S3.01 across all three G protein subtypes (Figure 5b). Therefore, on the ICL2 interface, more HBs were observed in the H3R-Gi complex than the H3R-Gq/Gs, which might provide the basis for the Gi preference of H3R (Figure 5b).

The H4R-Gi model exhibited four HBs, which were established between T127^ICL2^ and E28 of the Gi αN helix, R123^34.54^ in ICL2 and T129 of the Gi α2-β4 loop, and A119^34.50^ in ICL2 and N347 of the Gi α5 helix (Figure 5b). In addition, T124^34.55^ and V120^34.51^ of ICL2 formed HIs with R32 and L194 of Gi (Figure 5b). In the H4R-Gq model, H126^ICL2^ and R32 of the Gq αN helix formed an HB (Figure 5b). In the H4R-Gs model, T127^ICL2^ and R38 of the Gs αN helix formed an HB (Figure 5b). A119^34.50^ of H4R established an HI with the isoleucine in position G.H5.15 in the H4R-Gq/Gs models (Figure 5b,d). In conclusion, the H4R-Gi complex obsessed more HBs and HIs on the ICL2 interface than the H4R-Gq/Gs, which was consistent with the observation that H4R preferred the signaling to Gi over Gq/Gs (Figure 5b).

Therefore, the HBs and HIs formed by the ICL2 might be the critical determinants for the G protein bias among all four HRs. The ICL2 regions of the predicted models were largely confident. From the predicted models, the ICL2 exhibited more HBs and HIs in the primary engaged G proteins among HRs. Moreover, the C-terminus of the α5 helix exhibited more HBs and the N-terminus of the α5 helix displayed more HIs in the predicted models. The synergistic approach combining computational models and experimental structures facilitates the comprehensive understanding of GPCR modulation.

## 3. Discussion

Histamine receptors represent a critical neurotransmitter receptor system and are well-established targets for various agents used for the treatment of allergies, gastric ulcers, neurologic disorders, and inflammation. Previously determined cryo-EM structures of H4R have revealed significant disparities in the ligand binding and G protein interactions. These inconsistencies complicated the understanding of the mechanisms for the ligand-binding and G protein signaling among different HRs, posing challenges to HR-targeted therapeutic development.

In this study, we determined a new cryo-EM structure for the H4R-histamine-Gi complex at 2.9 Å resolution. By comparing our structure with the previously determined HR structures, we proposed distinct mechanisms for histamine recognition and G protein signaling. The H1R/H2R and H3R/H4R adopted opposite orientations of histamine, largely owing to site 5.46. The negatively charged Glu^5.46^ in H3R/H4R provided a stronger attraction to the cationic primary amine moiety of histamine over Asn^5.46^ and Thr^5.46^ in H1R/H2R, thereby stabilizing the reverse direction of histamine. Additionally, site 5.46 was generally occupied by the uncharged residues Ser^5.46^ or Ala^5.46^ in other monoamine receptors, where their agonists exhibited an H1R/H2R-like binding pattern [41,42,43]. This unique selection among HRs indicated a significant role of position 5.46 in histamine binding (Figure S3). By comparing the determined H4R-Gq/Gi structures, more interactions were seen at the H4R-Gi interface, especially the extensive hydrophilic interactions and HIs of TM5/6 and the Gi α5 helix, which might result in the preference of Gi over Gq for H4R.

Based on our current research, Y^6.51^ and F^7.39^ were identified as the specific probes for the histamine binding in H1R/H2R by HBs and H3R/H4R by π-π interactions, respectively. The HRs and muscarinic acetylcholine receptors possess tyrosine at position 6.51, while F^6.51^ is commonly used in the other aminergic receptors (Figure S3). In the dopamine D1R and serotonin 5HT_1A_R, F^6.51^ was observed to form π-π interactions or HIs with their agonists [49,56,57], but no HBs as in the H1R/H2R. Moreover, F^7.39^ was infrequently observed across the aminergic receptor family, being preferred only by the adrenergic receptors _α1A_AR-_α1D_AR and _α2A_AR-_α2C_AR (Figure S3). T^7.39^ in the dopamine D1R and L^7.39^ in the serotonin 5HT_1A_R generally established HIs with their agonists [49,56,57], while in H3R/H4R, π-π interactions and HBs were found between F^7.39^ and the imidazole ring of histamine. Together, these findings suggested positions 6.51 and 7.39 played notable roles in HRs and might be used for selective drug design targeting HRs. Additionally, Q^7.42^ in H4R played an important role in histamine binding and receptor activation, which contributed to the unique horizontal configuration and upward movement of the toggle switch in H4R, which might induce the constitutive activation of H4R [58].

Protein engineering is necessary for cryo-EM structure determination, including mutations, chimera G proteins, dominant negative Gi/Gs, and NanoBiT strategies, which might induce some variations in the receptor-ligand-transducer interactions. Recently, AF3 has been frequently used to predict the protein complexes using the wild-type sequences. In this study, we applied AF3 to predict 12 complexes of the four HRs with the three G protein subtypes. We found that the dominant G protein subtypes exhibited more interactions with ICL2, thus inducing G protein bias. Similar observations on the intracellular loops, particularly ICL2 and ICL3, determining the G protein preference in many other GPCRs have been extensively discussed [53,59,60,61]. The intensity of interactions between the receptors and transducers, specifically the distinct binding patterns of the α5 helix and αN helix of the G proteins to receptors, which were characterized by variations in the protrusion depth and angle relative to the receptors, determine the relative positions of the intracellular loops, thereby ultimately influencing the G protein bias [27]. Our findings thus shed light on the non-canonical activation mechanism of H4R and enhance our understanding of the specific signaling pathways within the histamine receptor family.

AF3 demonstrates substantial advances over AF2 in backbone accuracy, particularly enabling reliable de novo modeling of the GPCR architectures in data-scarce scenarios including the apo states and non-canonical GPCR assemblies. However, AF3 predictions tend to generate ensemble averages derived from its training data, whereas cryo-EM resolves specific conformational states stabilized by experimental conditions (e.g., protein constructs, nanobody engagement) that frequently induce conformational variations at the interfacial side chains [62,63]. Current limitations persist in AF3′s ability to accurately predict the ligand pharmacophore binding poses and LBP’s sidechain packings [34]. Nevertheless, AF3 establishes new benchmarks for AI-driven structural prediction, particularly advancing GPCR modeling [33]. These computational constraints reflect the intrinsic conformational plasticity of GPCRs, necessitating continued experimental validation. The synergistic integration of AF3′s predictive power with the cryo-EM/X-Ray-derived structures creates a multi-scale validation framework for deciphering GPCR activation landscapes. This paradigm shift accelerates targeted drug discovery by enabling the virtual screening and rational optimization of subtype-selective compounds.

Blocking the H4R signaling has been considered a potential treatment for chronic inflammatory diseases (CIDs) [14,15,16]. Emerging evidence showed that H4R antagonist JNJ-7777120 has an anti-inflammation effect while exhibiting partial agonistic activity in β-arrestin 2 recruitment [64,65]. Several cryo-EM structures of H4R bound to the agonists have been determined [13,26,27]. However, the H4R-antagonist structure is still elusive, limiting antagonist optimization. Determining the inactive structure of H4R represents a promising area for future research, by identifying the critical sites for the antagonist binding and selectivity.

In conclusion, a comparison of the H4R-G protein complexes reveals structural plasticity in the histamine binding modes and G protein-coupling interfaces. Crucially, we established a classification of monoamine conformations where the electrostatic properties of position 5.46 dictate the bifurcation into the H1R/H2R versus H3R/H4R. Through integrative experimental structures with computational models, we identified the critical roles of Y^6.51^ and Q^7.42^ in HR activation, characterized H4R’s unique toggle switch conformation, and demonstrated that ICL2 is a modulator of G protein bias. These findings provide a mechanistic framework for H4R histamine binding and signal transduction while revealing conserved/distinct pharmacological hotspots. The resolved structural nuances, particularly the dynamic HB networks and imidazole positioning variability, directly enable structure-based strategies to develop selective modulators targeting the subtype-specific conformational signatures through HB mimicry, advancing precision therapeutics for histaminergic disorders.

## 4. Materials and Methods

### 4.1. Protein Engineering

To facilitate expression, purification, and cryo-EM structure determination, human H4R was generated with several modifications. Maltose-binding protein (MBP) epitope was fused into the N terminus of H4R with truncations of the ICL3 residues 219–186 and C terminal residues 381–390, as the tedious ICL3 and C terminal loop would augment the instability of the receptor during the purification and cryo-EM processes. The chimeric H4R sequence was then subcloned into a modified pFastBac1 plasmid (Invitrogen, Waltham, MA, USA) with an N-terminal hemagglutinin (HA) signal peptide, followed by a FLAG tag, and a 10× His tag. The tobacco etch virus (TEV) protease cleavage site and linker residues (GSG) were inserted between MBP and H4R for the convenient MBP removal in the following purification process, as MBP epitope was used to increase the expression but often induced coagulation during concentration (Figure S1a). Human Gαi1 with four dominant-negative mutations (S47N, G203A, E245A, A326S; DNGαi) [49], which improved the stability of receptors and Gi, and human wild-type Gβ1 and Gγ2 [49,57] were cloned into the pFastBac1 vector [49,57] and co-expressed in *Spodoptera frugiperda* (Sf9) insect cells, together with a single-chain antibody ScFv16 [66].

### 4.2. Purification of H4R-Histamine-Gi Complex

Cell pellets were thawed at room temperature and lysed by dounce homogenization in lysis buffer (20 mM HEPES pH 7.5, 50 mM NaCl, 2 mM MgCl_2_), supplemented with Protease Inhibitor Cocktail (500 μM AEBSF, 1 μM E-64, 1 μM Leupeptin, and 150 nM Aprotinin). After centrifugation at 30,000× *g* for 30 min, membranes were resuspended in lysis buffer, containing 10 μM histamine (MCE), 100 mU/mL apyrase (NEB), and 2 mM β-mercaptoethanol (β-ME), followed by incubation at room temperature to form the complex. The washed membranes were solubilized with 0.5% (*w*/*v*) lauryl maltose neopentyl glycol (LMNG, Anatrace, Lucas, OH, USA), and 0.05% (*w*/*v*) cholesteryl hemisuccinate Tris salt (CHS, Anatrace, Lucas, OH, USA) at 4 °C for 3 h. The solubilized material was cleared by centrifugation and incubated with TALON (Takara, Kusatsu, Japan) resin overnight. After binding, the resin was packed into a gravity column (Bio-Rad, Hercules, CA, USA) and washed with 20 column volumes of buffer A consisting of 20 mM HEPES pH 7.5, 100 mM NaCl, 2 mM MgCl_2_, 0.01% (*w*/*v*) LMNG, 0.002% (*w*/*v*) CHS, and 10 μM histamine, containing 10 mM imidazole. The complex was eluted with 5 column volumes of buffer A containing 300 mM imidazole, 100 mU/mL apyrase, 2 mM β-ME, and 50 μM histamine. After TEV digestion overnight, the protein was subjected into a Superdex 200 Increase 10/300 column (GE Healthcare, Cleveland, OH, USA) pre-equilibrated with buffer containing 20 mM HEPES pH 7.5, 100 mM NaCl, 0.006% (*w*/*v*) glyco-diosgenin (GDN, Anatrace, Lucas, OH, USA), and 1 μM histamine. Peak fractions for the monomeric H4R-histamine-Gi complex were pooled and concentrated to ~5 mg/mL for the cryo-EM experiments. A schematic figure explaining the purification workflow is displayed in Figure S1b. The purity and homogeneity of the protein complex are shown in Figure S1c,d. The final yields of the purified complexes were ~0.3 mg/L insect cell culture.

### 4.3. Cryo-EM Grid Preparation and Data Collection

For cryo-EM grids preparation, 3 μL of purified H4R-histamine-Gi complex solution was applied onto glow-discharged holey carbon grids (Quantifoil Au R1.2/1.3, Jena, Germany). The grids were plunge-frozen into liquid ethane using FEI Vitrobot Mark IV (Thermo Fischer Scientific, Waltham, MA, USA) and stored in liquid nitrogen for subsequent data collection. Cryo-EM data collection was operated on a 300 kV Titan Krios electron microscope (Thermo Fisher Scientific) equipped with a GIF energy filter and a Falcon 4 direct electron detector (Thermo Fisher Scientific), at a magnification of 105,000× *g* in the Center of Cryo-Electron Microscopy (CCEM), Zhejiang University (China), corresponding to a nominal pixel size of 0.93 Å. Movies were recorded using EPU 4.0 software in counting mode at an exposure time of 4.51 s with a defocus range of −0.7 to −1.6 μm. A total of 5070 movies were collected for H4R-histamine-Gi, yielding high-quality datasets for further processing.

### 4.4. Cryo-EM Data Processing

The flow charts of data processing are presented in Figure S2. Dose-fractionated image stacks were aligned using MotionCor2 [67], and contrast transfer function (CTF) parameters were estimated using CTFFIND4.1 [68]. Subsequent image processing steps were carried out using RELION 4.0 [69]. Combined Laplacian-of-Gaussian-based auto-picking, Topaz picking, template pinking methods implemented in Relion 4.0 and cryoSPARC v3.3.2 [70]. A total of 3,935,936 particles were picked from 4841 movies. Through iterative 2D and 3D classification, 465,472 high-quality particles were selected and then subjected to Ab Initio Reconstruction into three classes. Rounds of Hetero-refinement were then performed and finally, one class displayed the complete H4R-Gi complex. Particles from this class were extracted and further refined through 3D classification, Bayesian polishing and CTF refinement in RELION 4.0 [71], yielding 261,176 in all. The final non-uniform (NU) refinement step generated a cryo-EM map with a global resolution of 2.91 Å, as determined by Fourier shell correlation at the 0.143 threshold. Local resolution estimations were calculated using cryoSPARC.

### 4.5. Model Building and Refinement

Cryo-EM structure models were built using the cryo-EM structure of the H4R-histamine-Gi complex (PDB ID: 8YN9) as the initial templates [72,73]. The models were docked into the EM density maps using UCSF Chimera and manually adjusted using COOT [74]. Refinement and validation were performed with Phenix [75,76] to optimize the geometry and fit to the density. Validation of the final models was conducted using Phenix validation tools, with detailed refinement statistics provided in Table S2. All structural figures were prepared using UCSF Chimera [77].

### 4.6. Structure Prediction

For structure predictions, we utilized the AF3 server (https://alphafoldserver.com/, accessed on 6 November 2024). The sequences submitted to the server corresponded to the UniProt entries (H1R: P35367, H2R: P25021, H3R: Q9Y5N1, H4R: Q9H3N8, Gαq protein: P50148, Gαi1 protein: P63096, Gαs protein: Q5JWF2, Gβ1: P62873, Gγ2: P59768).

For protein or protein complex prediction, only the protein sequences are required. Generally, we chose the entity type as protein. For apo GPCRs, only the GPCR sequences were submitted. For HR–G protein complexes, the sequence of each subunit was submitted individually as a single molecule, then all molecules were in total predicted. For each case, such as isolated H1R, or the H1R-Gq complex, we only predicted once, and the random seed was set to its default state, which reflected the prediction job provided in Figure S4. AlphaFold Server produces five predictions per job, and five models are ranked from 0 to 4, where 0 has the highest confidence as AF3 server Guides indicated (https://golgi.sandbox.google.com/guides#section-3:-interpreting-results-from-alphafold-server, accessed on 7 November 2024). Thus, the highest confidence which ranked 0 was selected as our model for further comparative analysis and plotting.

## Figures and Tables

**Table 1:** The global RMSDs of apo HRs between AlphaFold3 models and experimental structures.

Protein	State	pTM	pLDDT of LBP	PDB	AF3 Global RMSD (Å)
H1R	apo	0.63	>90	3RZE	0.566
H2R	apo	0.77	>90	7UL3	1.127
H3R	apo	0.69	largely >90	7F61	1.062
H4R	apo	0.72	-	-	-

A pTM score above 0.5 indicates the overall predicted fold for the complex might be similar to the true structure. A pLDDT score >90 stands for both the main chains and side chains predicted with high precision. The two structures are very similar when the RMSD is less than 1 Å generally.

**Table 2:** The global RMSDs of HRs between the AlphaFold3 models and cryo-EM structures.

Protein	State	ipTM	pTM	pLDDT of ICL2	PDB	AF3 Global RMSD (Å)
H1R-Gi	active	0.67	0.69	70–90		
H1R-Gq	active	0.71	0.71	70–90	7DFL	0.669
H1R-Gs	active	0.68	0.69	70–90		
H2R-Gi	active	0.74	0.75	70–90		
H2R-Gq	active	0.74	0.77	70–90		
H2R-Gs	active	0.79	0.76	>90	8YN3	0.749
H3R-Gi	active	0.73	0.73	70–90	8YN5	0.801
H3R-Gq	active	0.68	0.73	largely 70–90		
H3R-Gs	active	0.70	0.70	largely 70–90		
H4R-Gi	active	0.71	0.72	largely 70–90	8YN9	1.191
H4R-Gq	active	0.70	0.74	largely 70–90	7YFC	1.048
H4R-Gs	active	0.73	0.72	largely 70–90		

A pTM score above 0.5 indicates the overall predicted fold for the complex might be similar to the true structure; ipTM scores below 0.6 suggest a failed prediction; pLDDT scores between 70–90 are confident, and >90 are very highly confident; The two structures are very similar when the RMSD value is less than 1 Å, generally.

## Data Availability

Cryo-EM maps have been deposited in the Electron Microscopy Data Bank under accession codes: EMD-62803. The atomic coordinates have been deposited in the Protein Data Bank under accession code: 9L42. Source data are provided in this paper.

## Supplementary Materials

The following supporting information can be downloaded at: https://www.mdpi.com/article/10.3390/ph18030292/s1, Figure S1: Construct design and purification of H4R-histamine-Gi complex; Figure S2: H4R-histamine-Gi cryo-EM data processing and density map; Figure S3: Alignment of the ligand-contacting residues in human aminergic receptors; Figure S4: Models of predicted apo state H1R-H4R, and Gi-, Gq-, and Gs-coupled complexes by AF3; Figure S5: Confidence of predicted apo state H1R-H4R, and Gi-, Gq-, and Gs-coupled complexes by AF3; Table S1: Abbreviations and terms; Table S2: Cryo-EM data collection and refinement statistics of the H4R-histamine-Gi structure.
