# Supplementary material for: Cryo-EM Structures and AlphaFold3 Models of Histamine Receptors Reveal Diverse Ligand Binding and G Protein Bias
Source: Pharmaceuticals (Basel). 2025 Feb 21;18(3):292. doi: 10.3390/ph18030292 (PMC11946611; doi:10.3390/ph18030292)
Supplement: Supplementary file 1 [file pharmaceuticals-18-00292-s001.zip › pharmaceuticals-3420432-supplementary.pdf]

Supplementary Information

Cryo-EM structures and AlphaFold3 models of histamine receptors reveal diverse ligand binding and G proteins bias

Anqi Chen, Chenxi Su, Zisu Zhang, Haitao Zhang*

The Second Affiliated Hospital of Zhejiang University School of Medicine, Research Center for Clinical Pharmacy, Key Laboratory of Neuropharmacology and Translational Medicine of Zhejiang Province, State Key Laboratory of Advanced Drug Delivery and Release Systems, Institute of Pharmacology and Toxicology, College of Pharmaceutical Sciences, Zhejiang University, Hangzhou, Zhejiang 310058, China; anqichen@zju.edu.cn (A.C.); chenxisu@zju.edu.cn (C.S.); zhangzisu@zju.edu.cn (Z.Z.)

* Correspondence: haitaozhang@zju.edu.cn (H.Z.)

Table of contents

Entry	Section	Page
Figure S1	Construct design and purification of H4R-histamine-Gi complex.	S2
Figure S2	H4R-histamine-Gi complex cryo-EM data processing and density map.	S3
Figure S3	Alignment of the ligand-contacting residues in human aminergic receptors.	S4
Figure S4	Models of predicted apo state H1R-H4R, and Gi-, Gq-, and Gs-coupled complexes by AF3.	S5
Figure S5	Confidence of the predicted models of apo H1R-H4R, and Gi-, Gq-, and Gs-coupled complexes by AF3.	S6
Table S1	Abbreviations and terms	S7
Table S2	Cryo-EM data collection and refinement statistics of the H4R-histamine-Gi complex structure.	S8

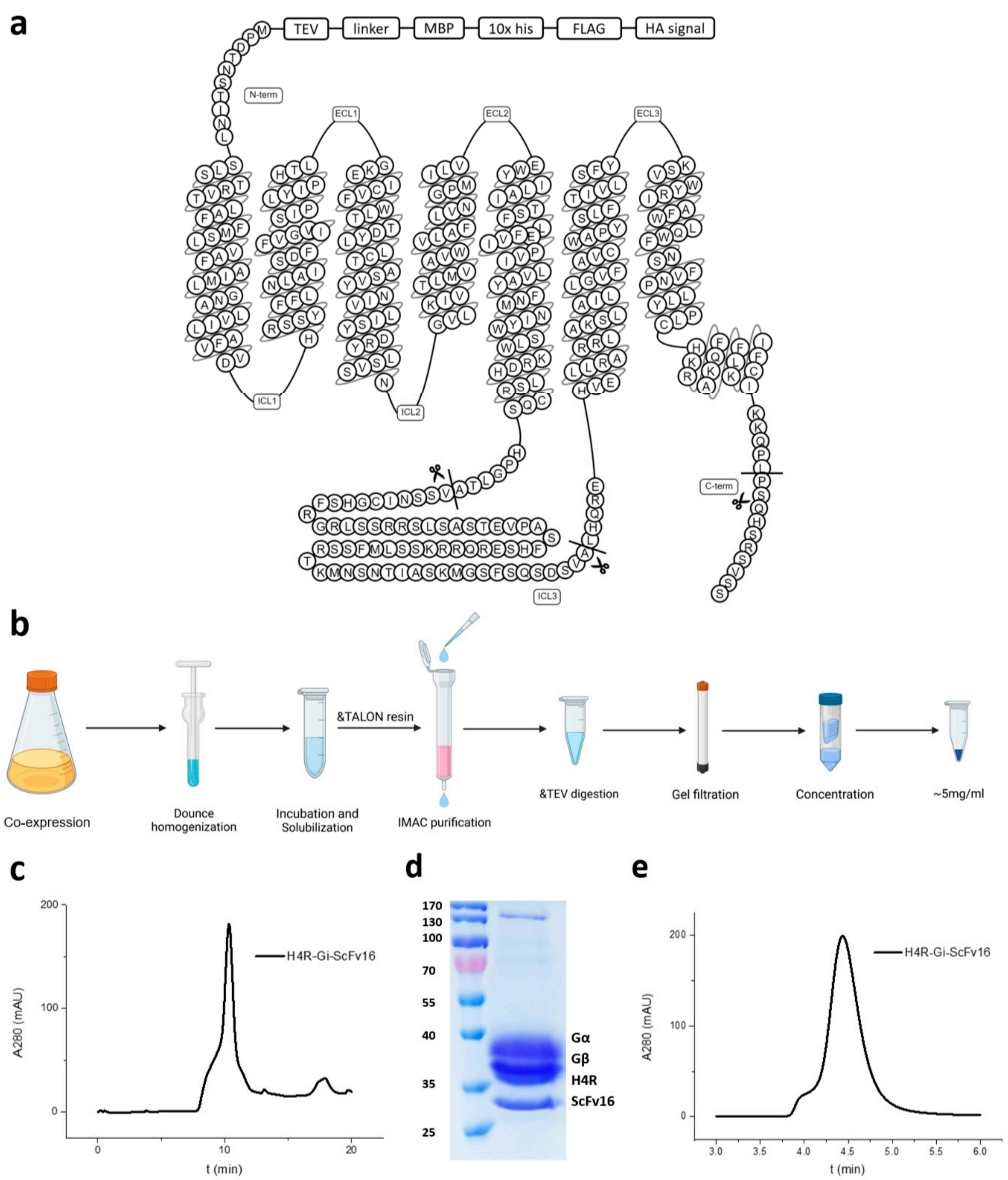

Figure S1. Construct design and purification of H4R-histamine-Gi complex. (**a**) Snake-shape diagram of the H4R construct used in complex assembling from GPCRdb (https://www.gpcrdb.org). (**b**) The schematic workflow of purification process. (**c-e**) Size exclusion chromatography (SEC) profile, SDS-PAGE, and analytic SEC of the H4R-Gi complex proteins.

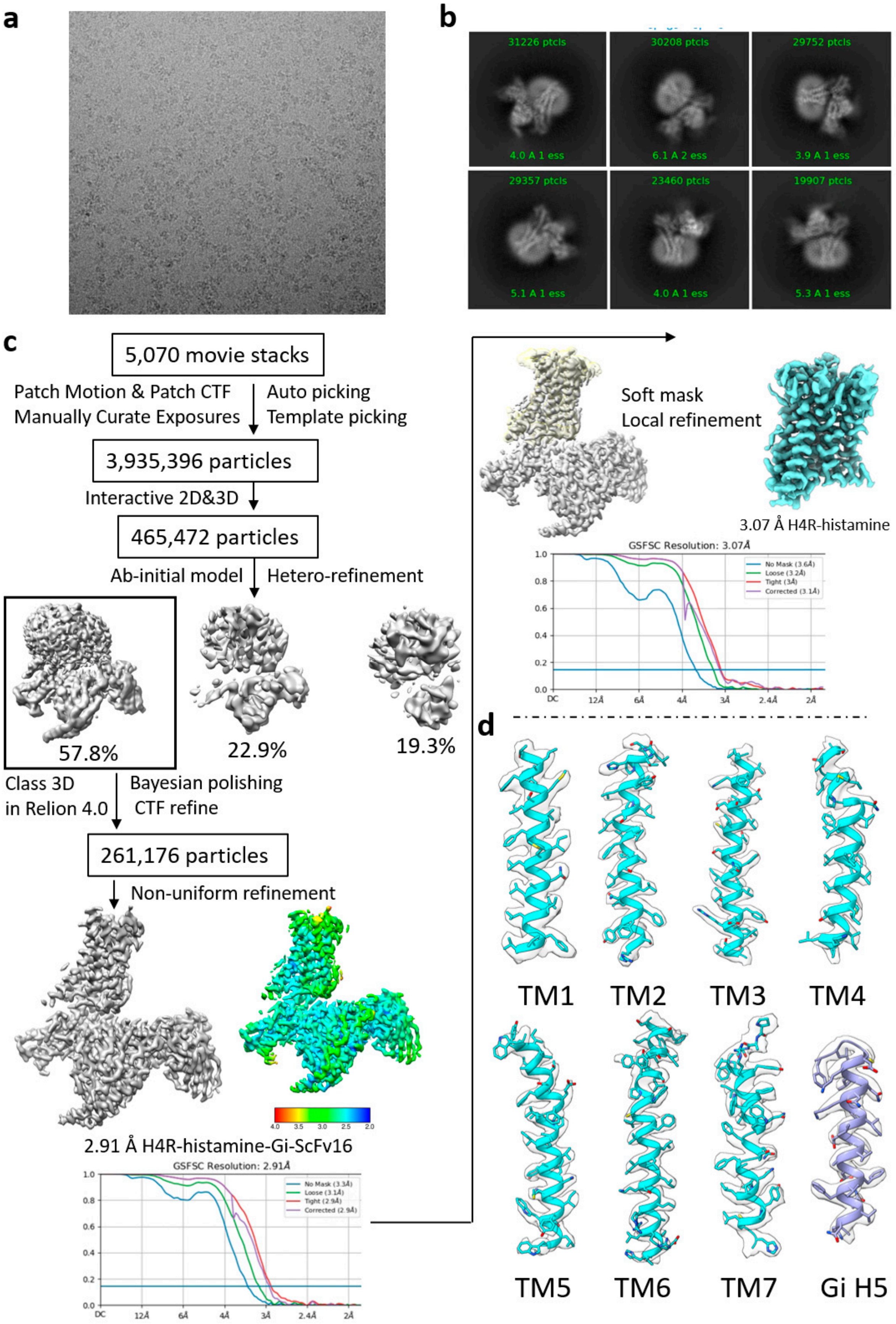

Figure S2. H4R-histamine-Gi complex cryo-EM data processing and density map. (**a**, **b**) Representative cryo-EM micrographs and 2D classification averages of the histamine-bound H4R-Gi. (**c**) Cryo-EM data processing workflows of the histamine-bound H4R-Gi. (**d**) Cryo-EM density maps of TM1-7, and α5 helix of Gαi in the histamine-H4R-Gi structure.

	2.61	2.64	2.65	3.28	3.29	3.32	3.33	3.36	3.37	3.40	34.50	34.51	34.52	34.53	34.54	34.55	34.56	34.57	34.58	5.38	5.39	5.42	5.43	5.46	5.47	6.44	6.48	6.51	6.52	6.55	6.56	6.58	6.59	7.32	7.35	7.36	7.39	7.40	7.42	7.43
HRH1	N	Y	L	W	L	D	Y	S	T	I	P	L	R	Y	L	K	Y	R	_	F	K	T	A	N	F	F	W	Y	F	F	M	I	A	E	H	M	I	W	G	Y
HRH2	S	Y	Q	Y	T	D	V	C	T	I	P	L	R	Y	P	V	L	V	_	Y	G	D	G	T	F	F	W	Y	F	F	V	R	G	E	E	A	L	W	G	Y
HRH3	Y	Y	V	W	L	D	Y	C	T	A	A	V	S	Y	R	A	Q	Q	G	F	L	A	S	E	F	F	W	Y	T	M	I	R	A	D	Y	E	F	W	L	W
HRH4	Y	H	T	W	L	D	Y	C	T	V	A	V	S	Y	R	T	Q	-	-	I	L	T	S	E	F	F	W	Y	S	T	I	L	S	S	Y	R	F	W	Q	W
5HT1A	A	Y	Q	F	I	D	V	C	T	I	P	I	D	Y	V	N	K	R	_	Y	T	S	T	A	F	F	W	F	F	A	L	L	P	T	G	A	N	W	G	Y
5HT1B	S	Y	T	W	L	D	I	C	T	I	A	V	E	Y	S	A	K	R	_	Y	T	S	T	A	F	F	W	F	F	S	L	M	P	L	F	D	T	W	G	Y
5HT1D	S	Y	T	W	L	D	I	C	T	I	A	L	E	Y	S	K	R	R	_	Y	T	S	T	A	F	F	W	F	F	S	L	L	P	P	F	D	T	W	G	Y
5HT1E	S	Y	I	W	L	D	M	C	T	I	A	I	E	Y	A	R	K	R	_	Y	T	S	T	A	F	F	W	F	F	E	L	V	G	S	A	D	T	W	G	Y
5HT1F	S	Y	I	W	L	D	I	C	T	I	A	V	E	Y	A	R	K	R	_	S	T	S	T	A	F	F	W	F	F	E	L	V	N	E	S	N	A	W	G	Y
5HT2A	S	T	I	W	I	D	V	S	T	I	P	I	H	H	S	R	F	N	_	F	V	G	S	S	F	F	W	F	F	N	I	A	V	G	L	N	V	W	G	Y
5HT2B	A	T	I	W	L	D	V	S	T	I	P	I	Q	A	N	Q	Y	N	_	F	M	G	S	A	F	F	W	F	F	N	I	L	V	Q	L	E	V	W	G	Y
5HT2C	S	A	I	W	I	D	V	S	T	I	P	I	E	H	S	R	F	N	_	F	V	G	S	A	F	F	W	F	F	N	I	S	V	E	L	N	V	W	G	Y
5HT4R	G	E	L	R	T	D	V	T	T	I	P	L	V	Y	R	N	K	M	_	Y	A	C	S	A	F	F	W	F	F	N	I	D	P	G	W	T	L	W	G	Y
5HT5A	S	H	E	W	I	D	V	C	T	I	H	M	E	Y	T	_	_	_	_	Y	A	S	T	A	F	F	W	F	F	E	L	S	P	A	K	S	L	W	G	Y
5HT6R	A	N	A	W	T	D	V	C	S	I	P	L	R	Y	K	L	R	M	_	F	V	A	S	T	F	F	W	F	F	N	I	Q	A	P	F	D	T	W	G	Y
5HT7R	V	T	D	F	I	D	V	C	T	I	P	L	T	Y	P	V	R	Q	_	Y	T	S	T	A	F	F	W	F	F	S	T	R	P	L	E	R	L	W	G	Y
ACM1	Y	Y	L	W	L	D	Y	S	N	V	P	L	S	Y	R	A	K	R	_	I	T	T	A	A	F	F	W	Y	N	V	L	S	T	E	W	E	Y	W	C	Y
ACM2	Y	Y	T	W	L	D	Y	S	N	V	P	L	T	Y	P	V	K	R	_	V	T	T	A	A	F	F	W	Y	N	V	L	N	T	N	W	T	Y	W	C	Y
ACM3	F	Y	I	W	L	D	Y	S	N	V	P	L	T	Y	R	A	K	R	_	I	T	T	A	A	F	F	W	Y	N	V	L	N	T	K	W	N	Y	W	C	Y
ACM4	Y	Y	I	W	L	D	Y	S	N	V	P	L	T	Y	P	A	R	R	_	V	T	T	A	A	F	F	W	Y	N	V	L	N	T	D	W	S	Y	W	C	Y
ACM5	Y	Y	I	W	L	D	Y	S	N	V	P	L	T	Y	R	A	K	R	_	I	T	T	A	A	F	F	W	Y	N	V	L	S	T	V	W	H	Y	W	C	Y
ADA1A	S	F	E	W	A	D	V	C	T	I	P	L	R	Y	P	T	I	V	_	Y	V	S	A	S	F	F	W	F	F	M	P	G	S	E	F	K	F	W	G	Y
ADA1B	S	L	E	W	A	D	V	C	T	I	S	L	Q	Y	P	T	L	V	_	Y	A	S	S	S	F	F	W	F	F	L	P	G	S	D	F	K	F	W	G	Y
ADA1D	S	M	E	W	A	D	V	C	T	I	S	L	K	Y	P	A	I	M	_	Y	A	S	S	S	F	F	W	F	F	L	P	G	S	E	F	K	F	W	G	Y
ADA2A	S	N	E	Y	L	D	V	C	T	I	A	I	E	Y	N	L	K	R	_	Y	V	S	C	S	F	F	W	F	F	Y	T	T	A	R	F	K	F	W	G	Y
ADA2B	S	N	E	Y	L	D	V	C	T	I	A	L	E	Y	N	S	K	R	_	Y	I	S	S	S	F	F	W	F	F	Y	S	G	A	H	F	Q	F	W	G	Y
ADA2C	S	N	E	Y	L	D	V	C	T	I	A	V	E	Y	N	L	K	R	_	Y	I	S	C	S	F	F	W	F	F	Y	S	Y	G	G	F	K	F	W	G	Y
ADRB1	G	I	V	W	T	D	V	V	T	I	P	F	R	Y	Q	S	L	L	_	Y	A	S	S	S	F	F	W	F	F	N	V	K	A	D	F	V	N	W	G	Y
ADRB2	G	H	I	W	T	D	V	V	T	I	P	F	K	Y	Q	S	L	L	_	Y	A	S	S	S	F	F	W	F	F	N	I	H	V	K	Y	I	N	W	G	Y
ADRB3	A	L	A	W	T	D	V	V	T	I	P	L	R	Y	G	A	L	V	_	Y	V	S	S	S	F	F	W	F	F	N	V	R	A	G	F	L	N	W	G	Y
DRD1	K	A	E	W	V	D	I	S	T	I	P	F	R	Y	E	R	K	M	_	Y	A	S	S	S	F	F	W	F	F	N	C	L	P	S	F	D	V	W	G	W
DRD2	V	L	E	F	V	D	V	C	T	I	P	M	L	Y	N	_	_	_	_	F	V	S	S	S	F	F	W	F	F	H	I	N	I	P	Y	S	T	W	G	Y
DRD3	V	L	E	F	V	D	V	C	T	I	P	V	H	Y	Q	H	G	T	G	F	V	S	S	S	F	F	W	F	F	H	V	N	T	P	Y	S	T	W	G	Y
DRD4	F	S	E	L	M	D	V	C	T	I	P	L	R	Y	N	R	Q	G	_	Y	V	S	S	S	F	F	W	F	F	H	I	Q	A	P	V	S	T	W	G	Y
DRD5	K	A	E	W	V	D	I	S	T	I	P	F	R	Y	K	R	K	M	_	Y	A	S	S	S	F	F	W	F	F	N	C	V	P	E	F	D	V	W	G	W
TAAR1	S	R	S	H	T	D	I	S	S	I	P	L	R	Y	K	A	K	M	_	S	G	T	F	S	F	F	W	F	F	T	V	D	P	P	N	D	I	W	G	Y

Figure S3. Alignment of the ligand-contacting residues in human aminergic receptors. The residue positions are manifested by the Ballesteros-Weinstein numbers at the top line. The blue boxes stand for key residues for a more horizontal position of toggle switch in H3R and H4R. The positions of amino acids participated in ligand binding pocket (LBP) formation are depicted as yellow boxes. The green box is on behalf of the key driver dictating the electrostatic potential surface of LBP in histamine receptors. The residues which are colored in histamine family are not conserved in aminergic family.

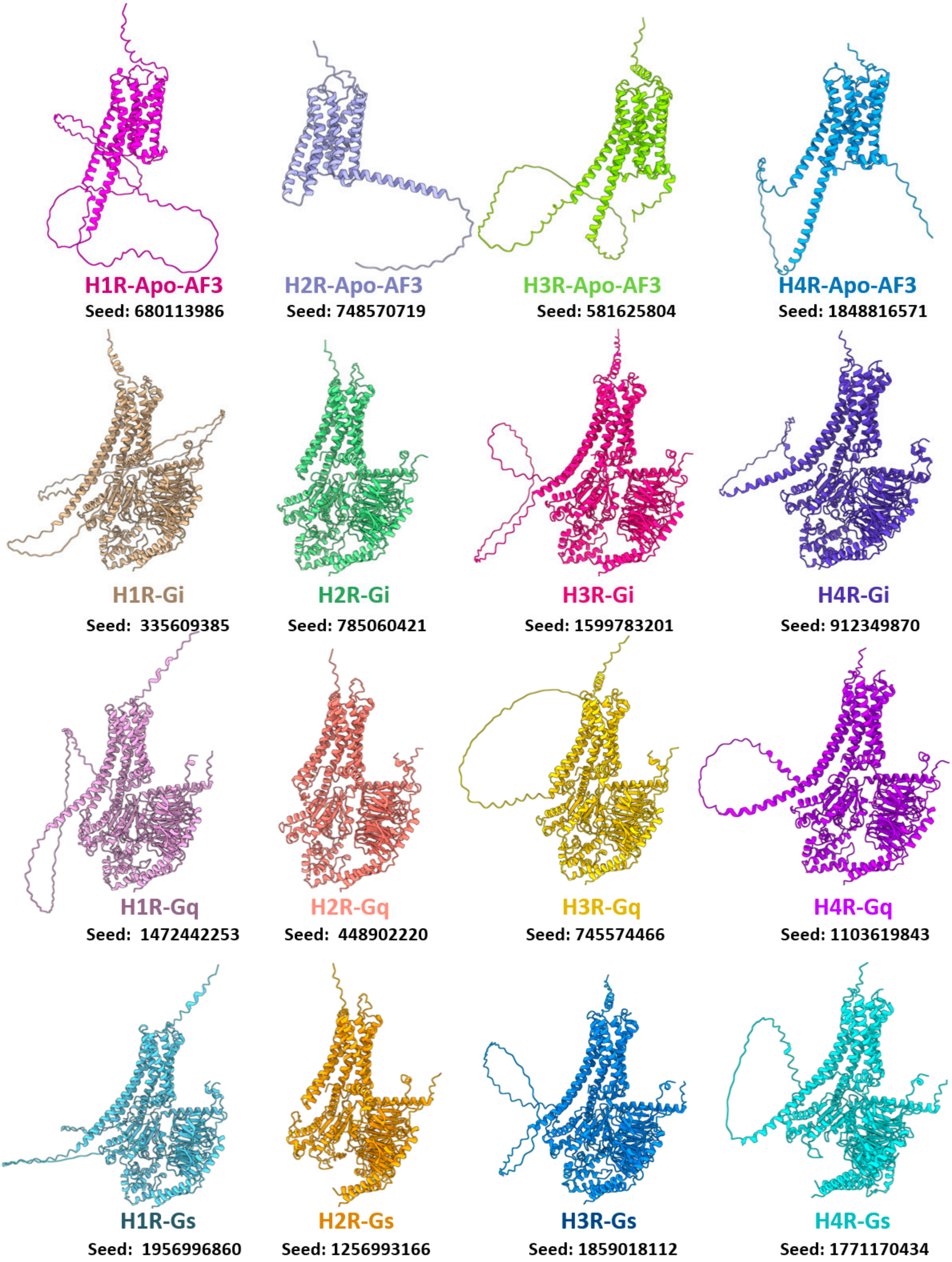

Figure S4. Models of predicted apo state H1R-H4R, and Gi-, Gq-, and Gs-coupled complexes by AF3 with according seeds respectively.

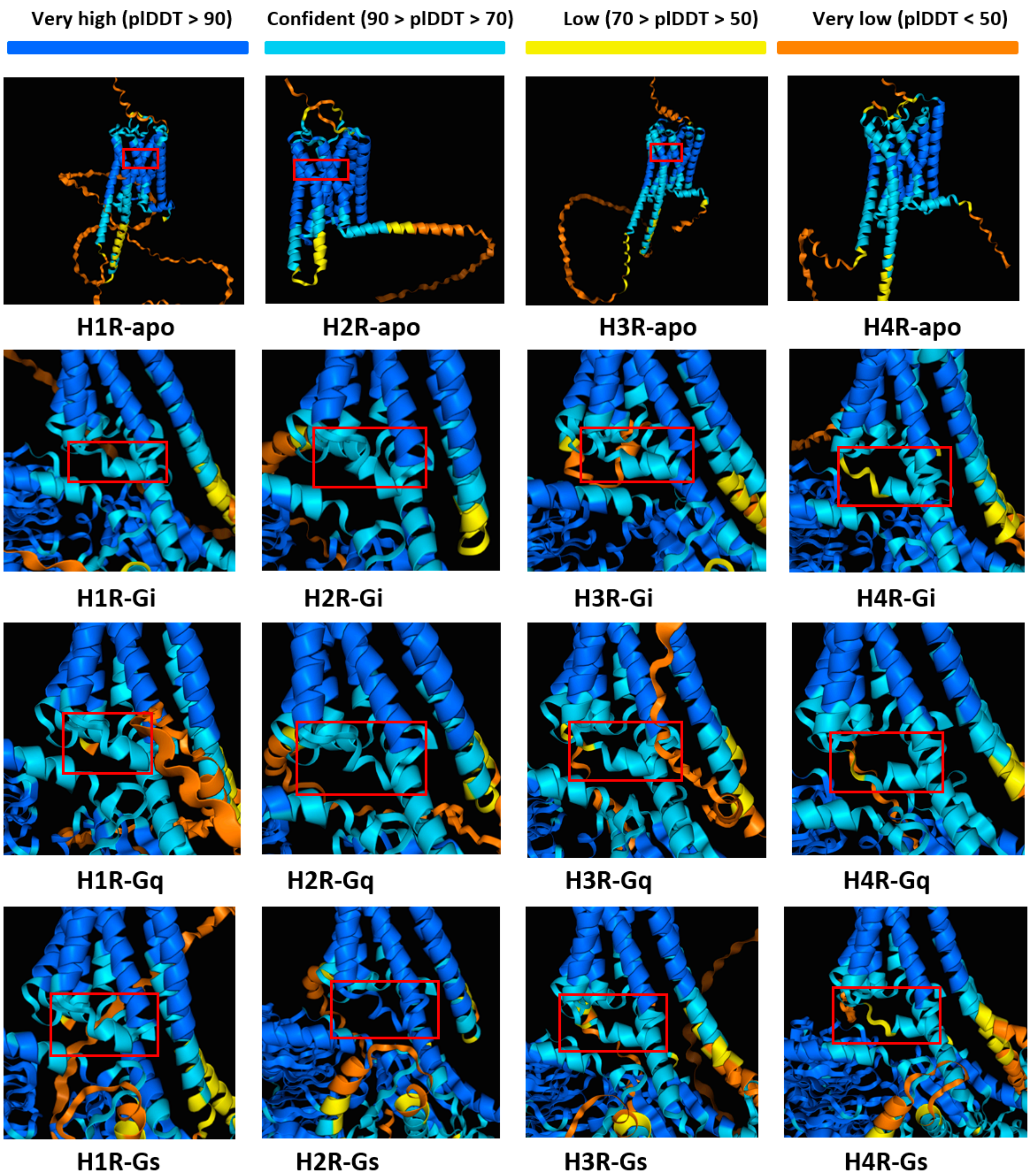

Figure S5. The confidence of the predicted models of apo H1R-H4R, and Gi-, Gq-, and Gs-coupled complexes by AF3 with according plDDT respectively. The ligand binding pocket (LBP) of apo histamine receptors (HRs) and intracellular loop 2 (ICL2) of HR-G proteins complexes were boxed in red. The boxed parts are very high confident or confident.

Table S1. Abbreviations and terms

Abbreviation	Full name
AF	AlphaFold
AI	Artificial intelligence
AT1R	Angiotensin II type 1 receptor
aSEC	Analytical size exclusion column
CHS	Cholesteryl hemisuccinate
CNS	Central nervous system
cryo-EM	Cryo-electron microscopy
CTF	Contrast transfer function
ECL	Extracellular loop
EMDB	Electron Microscopy Data Bank
GDN	Glyco-diosgenin
Gi	Inhibitory guanosine triphosphate binding protein
GPCR	G protein-coupled receptor
Gq	Guanosine triphosphate binding protein q
Gs	Stimulatory guanosine triphosphate binding protein
HB	Hydrogen bond
HI	Hydrophobic interaction
HR	Histamine receptor
H1R	Histamine 1 receptor
H2R	Histamine 2 receptor
H3R	Histamine 3 receptor
H4R	Histamine 4 receptor
ICL	Intracellular loop
LBP	Ligand-binding pocket
PDB	Protein data bank
RMSD	Root-mean-square deviation
TGF	transforming growth factor
TM	Transmembrane

Table S2. Cryo-EM data collection and refinement statistics of the H4R-histamine-Gi complex structure

	**H4R-histamine-Gi**
**PDB ID/EMDB ID**	PDB-9L42/EMD-62803
**Data collection and processing**	
Magnification	130k×
Voltage (kV)	300
Electron exposure (e-/Å^2)	50
Defocus range (μm)	-0.7~-1.5
Pixel size (Å)	0.93
Symmetry imposed	*C*1
Initial particle projections (no.)	3,935,396
Final particle projections (no.)	261,176
Map resolution (Å)	2.91
FSC threshold	0.143
Map resolution range (Å)	2.5-4.0
**Refinement**	
FSC threshold	0.5
Map sharpening *B* factor (Å^2)	-50
Model composition	
Non-hydrogen atoms	8,824
Protein residues	1,126
B factors (Å^2)	103.33
R.m.s.deviations	
Bond lengths (Å)	0.008
Bond angles (°)	0.946
Validation	
MolProbity score	1.55
Clash score	7.27
Rotamer outliers (%)	0.87
Ramachandran plot	
Favored (%)	97.20
Allowed (%)	2.80
Disallowed (%)	0
